# Supplementary material for: A novel multi-user collaborative cognitive radio spectrum sensing model: Based on a CNN-LSTM model
Source: PLoS One. 2025 Jan 15;20(1):e0316291. doi: 10.1371/journal.pone.0316291 (PMC11734992; doi:10.1371/journal.pone.0316291)
Supplement: Attachment — Submitted filename: review comments.docx [file pone.0316291.s002.docx]

**PAPER TITLE: A Novel Multi-User Collaborative Cognitive Radio 2 Spectrum Sensing Model: Based On A CNN-LSTM 3 model**

**Reviewer: Dr.P.Ezhumalai, Professor&Head, R.M.D Engineering College**

**Comments:**

- The research endeavors to address the intricate challenges that arise in dynamic spectrum environments through the strategic utilization of convolutional neural networks (CNNs) and long short-term memory networks (LSTMs).
- By synergizing the capabilities of CNN for robust feature extraction and LSTM for meticulous sequential data analysis, the approach adeptly captures the intricate spatial and temporal relationships inherent in spectrum sensing tasks, ultimately culminating in heightened precision and computational efficiency.
- Moreover, the collaborative multi-user framework substantially bolsters detection reliability by effectively mitigating the impact of individual user errors, thereby rendering this methodology exceptionally well-suited for deployment in practical cognitive radio networks.
- In essence, the study emerges as a comprehensive and technically proficient exploration, characterized by its profound impact on the realms of spectrum management and cognitive radio networks, thereby paving the way for significant advancements in the field.
